# Magnetic Resonance Imaging of Cerebral Aspergillosis: Imaging and Pathological Correlations

**DOI:** 10.1371/journal.pone.0152475

**Published:** 2016-04-20

**Authors:** Guillaume Marzolf, Marcela Sabou, Béatrice Lannes, François Cotton, David Meyronet, Damien Galanaud, Jean-Philippe Cottier, Sylvie Grand, Hubert Desal, Julie Kreutz, Maleka Schenck, Nicolas Meyer, Francis Schneider, Jean-Louis Dietemann, Meriam Koob, Raoul Herbrecht, Stéphane Kremer

**Affiliations:** 1 Département de Neuroradiologie, Hôpitaux Universitaires de Strasbourg, Strasbourg, France; 2 Laboratoire de Parasitologie et de Mycologie Médicale, Hôpitaux Universitaires de Strasbourg, Strasbourg, France; 3 Service de Pathologie, Hôpitaux Universitaires de Strasbourg, Strasbourg, France; 4 Département de Neuroradiologie, Centre Hospitalier Lyon Sud, Hospices Civils de Lyon, Lyon, France; 5 Département de Neuropathologie, Hospices Civils de Lyon, Lyon, France; 6 Département de Neuroradiologie, Hôpital de la Pitié Salpêtrière, Hôpitaux de Paris, Paris, France; 7 Département de Neuroradiologie, Centre Hospitalier Universitaire de Tours, Tours, France; 8 Département de Neuroradiologie, Centre Hospitalier Universitaire de Grenoble, Grenoble, France; 9 Département de Neuroradiologie, Hôpital Nord Laennec, Centre Hospitalier Universitaire de Nantes, Nantes, France; 10 Département de Radiologie, Centre Hospitalier Universitaire de Liège, Liège, Belgium; 11 Service de Réanimation Médicale, Hôpitaux Universitaires de Strasbourg, Strasbourg, France; 12 Laboratoire de Biostatistique, Faculté de Médecine de Strasbourg, Strasbourg, France; 13 Département d'Oncologie et d’Hématologie, Hôpitaux Universitaires de Strasbourg, Strasbourg, France; Shenzhen institutes of advanced technology, CHINA

## Abstract

Cerebral aspergillosis is associated with a significant morbidity and mortality rate. The imaging data present different patterns and no full consensus exists on typical imaging characteristics of the cerebral lesions. We reviewed MRI findings in 21 patients with cerebral aspergillosis and correlated them to the immune status of the patients and to neuropathological findings when tissue was available. The lesions were characterized by their number, topography, and MRI signal. Dissemination to the brain resulted from direct spread from paranasal sinuses in 8 patients, 6 of them being immunocompetent. Hematogenous dissemination was observed in 13 patients, all were immunosuppressed. In this later group we identified a total of 329 parenchymal abscesses involving the whole brain with a predilection for the corticomedullary junction. More than half the patients had a corpus callosum lesion. Hemorrhagic lesions accounted for 13% and contrast enhancement was observed in 61% of the lesions. Patients with hematogenous dissemination were younger (p = 0.003), had more intracranial lesions (p = 0.0004) and had a higher 12-week mortality rate (p = 0.046) than patients with direct spread from paranasal sinuses. Analysis of 12 aneurysms allowed us to highlight two distinct situations. In case of direct spread from the paranasal sinuses, aneurysms are saccular and located on the proximal artery portions, while the hematogenous dissemination in immunocompromised patients is more frequently associated with distal and fusiform aneurysms. MRI is the exam of choice for cerebral aspergillosis. Number and type of lesions are different according to the mode of dissemination of the infection.

## Introduction

Aspergillosis is a filamentous fungal infection transmitted by inhalation of airborne spores or by contamination of wounds [1]. This disease develops primarily in immunocompromised patients and may progress to disseminated aspergillosis defined by the infection of at least two noncontiguous organs. The brain is one of the most commonly affected secondary sites and is involved in 10–44% of patients [2–8]. Cerebral aspergillosis is the worst clinical form with a 85–100% mortality rate [9,10]. Despite the development of *Aspergillus* galactomannan and the beta-D-glucan detection tests as well as PCR, the mycological data are not always conclusive and imaging modalities such as CT-scan for invasive pulmonary aspergillosis (IPA) and CT combined with MRI for sinus and cerebral aspergillosis are crucial. A review of literature on the MRI appearances of cerebral aspergillosis shows that a few lesions are enhanced after gadolinium injection with a nodular or annular presentation (Table 1) [4–6,11–23]. As for other abscessed lesions, the most common pattern consists of a T1W hypointense signal and a T2W hyperintense signal. However, in the literature some presentations stand out, including mainly a T2 intermediate with surrounding high signal, a T1 hyperintense ring, and a target-like ADC (apparent diffusion coefficient) signal [4,12]. A few cases of mycotic aneurysms due to *Aspergillus* have also been reported [17,20]. The purpose of this study was to describe the variety of MRI presentation of cerebral aspergillosis precisely and to compare these presentations to pathological findings.

**Table 1:** Review of comparable studies.

STUDY	No. of patients / lesions	IS /mortality rate	Topography, comments	Hemorrhage	T2 Intermediate with surrounding high signal	Target-like ADC	Contrast-enhancement	Vascular complications
***Almutairi et al*. *2009***	1/>20	Y/100%	All areas including the brainstem but mostly seen at the CMJ.	Y	Y	1 lesion	Y	N
***Ashdown et al*. *1994***	11/*NA*	Y/*NA*	Multiple lesions predominant at the CMJ. One patient had nasosinusal involvement.	Y	*NA*	*NA*	4p	N
***Charlot et al*. *2007***	3/23	Y/100%	All areas (19 supratentorial, 4 infratentorial).	*NA*	Y	52% lesions	1p	N
***DeLone et al*. *1999***	18/*NA*	Y/89%	12 patients with MRI. Lesions involving BNT (13p), CMJ (10p), corpus callosum (7p), brainstem (2p).	8p	*NA*	*NA*	7p	N
***Dietrich et al*. *2001***	6/36	Y/100%	All areas but mostly seen at the CMJ.	39% lesions	*NA*	*NA*	42% lesions	N
***Gaviani et al*. *2005***	8/*NA*	Y/75%	6 Patients with *Aspergillus*. Lesions involving CMJ, BNT and white matter.	N	2p	1p	6p	N
***Gabelmann et al*. *2007***	9/*NA*	Y/89%	4 patients with MRI. Two patients with nasosinusal involvement.	1p	*NA*	*NA*	4p	ICA (proximal)
***Hurst et al*. *2001***	1/*NA*	Y/100%	No MRI performed. Nasosinusal involvement.	*NA*	*NA*	*NA*	*NA*	ICA (fusiform, proximal)
***Kami et al*. *1999***	1/*NA*	Y/100%	Multiple lesions located in the right cerebral hemisphere.	*NA*	*NA*	*NA*	*NA*	N
***Lee et al*. *2013***	1/*NA*	N/0%	Multiple lesions located in the CMJ and WM.	Y	*NA*	*NA*	Y	N
***Miaux et al*. *1995***	5/*NA*	Y/100%	All areas.	N	Y	*NA*	2p	N
***Negoro et al*. *2013***	1/0	Y/0%	No parenchymal lesion. Nasosinusal involvement.	*NA*	*NA*	*NA*	*NA*	ACA (saccular, proximal)
***Okafuji et al*. *2003***	1/*NA*	Y/100%	All areas but mostly seen at the CMJ.	Y	*NA*	*NA*	Y	N
***Pollack et al*. *2007***	1/1	N/100%	Lesion located at the CMJ of cerebrum.	Y	*NA*	*NA*	Y	N
***Tempkin et al*. *2006***	1/*NA*	Y/100%	No topographic description.	Y	Y	*NA*	Y	N
***Yamada et al*. *2002***	8/27	Y (50%)/≥75%	5 Patients with MRI. Lesions located in the CMJ, posterior fossa and BNT. One patient with meningitis.	25% lesions	*NA*	*NA*	55% lesions	N

Abbreviations.—IS: Immunosuppression; *NA*: not available; p: patient; CMJ: corticomedullary junction; BNT: basal nuclei and thalami; WM: white matter; ICA: internal carotid artery; ACA: anterior cerebral artery

## Patients and Methods

We retrospectively reviewed neuropathological, clinical and neuroimaging findings of 21 patients with cerebral aspergillosis. These cases were identified over a period of 8 years (from 2006 to 2013) and collected in seven French and Belgian university hospitals. The study was approved as a retrospective non-interventional study by the local ethics committee (Comité d'Ethique de la Faculté de Médecine, d'Odontologie, de Pharmacie, des Ecoles d'Infirmières, de Kinésithérapie, de Maïeutique et des Hôpitaux, Strasbourg). Two trained neuroradiologists analyzed imaging data to describe the radiological semiology of the different lesions. The results were compared to the patient’s pathological findings and immune status, in cooperation with a neuropathologist and a specialist in clinical mycology. The Fisher exact test and Mann-Whitney test were performed when appropriate using GraphPad Prism version 6.00 (GraphPad Software, La Jolla, CA). Diagnosis of invasive aspergillosis was based microscopy and culture of respiratory samples, microscopy, culture and histopathology of sinus, brain or other tissue biopsies or on positive *Aspergillus* galactomannan detection test in serum, CSF or bronchoalveolar lavage fluid. Tissues were stained by periodic acid-Schiff, hematoxylin-eosin and Grocott’s methenamine silver stain. Tissue samples came from surgical biopsy (*n* = 9) or necropsy (*n* = 6). For each case, the fungal infection by *Aspergillus* was classified as proven or probable according to a strict application of the 2008 EORTC/MSG definitions [24]. One patient (#11) had initially an IPA with a rapid dissemination to the brain documented by clinical signs and MRI and secondary presented also a *Trichosporon mycotoxinivorans* fungemia and ultimately an additional *Scedosporium apiospermum* disseminated infection [25]. This case was included in the study because the cerebral infection was documented while *A*. *fumigatus* was the only pathogen identified in this patient. Imaging studies were performed on 1.5- and/or 3-Tesla MRI. The investigations had to include at least T1-weighted and T2W sequences, fluid-attenuated inversion recovery sequences (FLAIR), gradient-echo T2*W, T1W sequences after gadolinium intravenous injection and DWI (diffusion-weighted imaging with b-values of 0 and 1000 s/mm^2^). Imaging information was gathered such as the number of lesions depending on the dissemination pathway, their topography and the signal characteristics. We based the investigation on the main MRI aspects such as a T1 hyperintense ring, T2 intermediate with surrounding high signal and, finally, the target-like ADC signal. Charlot *et al*. pointed out the presence of round these target-like lesions presenting central and peripheral hypointense areas on diffusion-weighted sequences, with high ADC values, and an intermediate striking hyperintense area on DWI with low ADC values [12,26].

## Results

Twenty-one cases appropriately explored by MRI were identified. Most patients were immunosuppressed (15/21, 71%), due to organ transplantation treatment for seven of them. A majority of the immunocompromised patients had primarily IPA (11/15). The patients’ sex, age, risk factors, immunological status, mycological positive findings, degree of certainty of the aspergillosis, organs involved, cerebral lesion characteristics, and outcome are detailed in Table 2.

**Table 2:** Characteristics of 21 patients with cerebral aspergillosis.

#/age/sex	Risk factors	IS	Mycological positive findings	Pathogen	Degree of certainty	Other organs involved	MRI findings	Survival, follow-up
1 /59/M	Sphenoidal sinusitis	No	Surgical debridement	*A*. *flavus*	Proven	Paranasal sinuses	Lateral sinus thrombophlebitis, aneurysms	Yes, 21 months
2 /73/M	Facial injury with osteosynthesis	No	Surgical resection and debridement	*A*. *fumigatus*	Proven	Paranasal sinuses	Abscess, subdural empyema	Yes, 4 years
3 /72/M	Ethmoidal sinusitis, multiple myeloma, steroids	Yes	Surgical resection	*Aspergillus* sp.	Proven	Paranasal sinuses, orbital cavity	Abscess	Yes, 14 months
4 /77/M	Ethmoidal sinusitis	No	Surgical resection	*A*. *fumigatus*	Proven	Paranasal sinuses	Aneurysm, abscesses, subdural empyema	Yes, 10 months
5 /55/F	Frontal sinusitis, lung Tx, T-cell suppressor, steroids	Yes	Surgical resection	*A*. *fumigatus*	Proven	Paranasal sinuses	Abscess	Yes, 5 years
6 /77/M	Facial injury with osteosynthesis	No	Surgical resection	*A*. *fumigatus*	Proven	Paranasal sinuses	Abscess	Yes, 23 months
7 /65/F	Frontal sinusitis, lymphoma in remission for 11 years	No	Surgical resection	*Septate hyphae*	Probable	Paranasal sinuses	Abscess	Yes, 12 months
8 /84/M	Sphenoidal sinusitis	No	Sinus biopsies	*A*. *fumigatus*	Proven	Paranasal sinuses, orbital cavity	Abscess, subdural empyema	Yes, 7 months
9 /74/M	Myeloid sarcoma, chemotherapy, neutropenia	Yes	Tracheal aspiration, GM (BAL, serum)	*A*. *ochraceus*	Proven	Lung	Abscess	No, 4 days
10 /64/M	Heart Tx, T-cell suppressor, steroids	Yes	GM (CSF, serum)	*Aspergillus* sp.	Probable	Lung	Abscesses	Yes, 5 years
11 /47/F	Crohn disease, steroids, acute liver failure	Yes	Sputum, tracheal aspiration, BAL, GM (BAL, serum)	*A*. *fumigatus*	Probable	Lung	Abscesses, aneurysm	No, 31 days
12 /35/M	Lung/liver Tx, T-cell suppressor, steroids, cystic fibrosis, diabetes mellitus	Yes	Sputum, tracheal aspiration, GM (serum), post-mortem biopsies (lung, trachea, bronchi, brain, heart, thyroid, liver, pancreas, small bowel)	*A*. *fumigatus*	Proven	Lung, trachea, bronchi, heart, thyroid, liver, pancreas, small bowel	Abscesses	No, 23 days
13 /44/M	Alcoholic liver failure, steroids, liver cirrhosis	Yes	Tracheal aspiration, post-mortem (thyroid, kidney, spleen, brain)	*A*. *fumigatus*	Proven	Lung, kidney, spleen	Abscesses, aneurysms	No, 20 days
14 /57/M	Lung Tx, T-cell suppressor, steroids	Yes	Tracheal aspiration, lung, skin, bronchus, post-mortem (kidney, heart, brain)	*A*. *fumigatus*	Proven	Lung, heart, skin	Abscesses	No, 11 months
15 /59/M	Glioblastoma, radio-chemotherapy	Yes	Post-mortem (lung, brain)	*A*. *fumigatus*	Proven	Lung	Abscesses	No, 83 days
16 /58/M	Heart Tx, T-cell suppressor, steroids	Yes	GM (CSF, BAL)	*Aspergillus* sp.	Probable	Lung	Abscesses	Yes, 3 years
17 /55/M	Liver Tx, T-cell suppressor, steroids	Yes	GM (CSF, BAL, serum)	*Aspergillus* sp.	Probable	Lung, heart	Abscesses, cerebral ischemia	Yes, 13 months
18 /53/M	Bladder neoplasm, chemotherapy, steroids	Yes	Sputum, GM (BAL)	*A*. *fumigatus*	Probable	Lung	Abscesses	No, 62 days
19 /49/M	AIDS	Yes	Post-mortem (brain)	*A*. *fumigatus*	Proven	-	Abscesses	No, 68 days
20 /58/F	End-stage kidney failure, hemodialysis, steroids	Yes	Tracheal aspiration, GM (serum), post-mortem (lung, heart, thyroid, lymph node, brain)	*A*. *nidulans*	Proven	Lung, heart, thyroid, lymph node	Abscesses	No, 18 days
21 /58/M	Heart Tx, T-cell suppressor, steroids	Yes	Brain biopsy	*A*. *fumigatus*	Proven	-	Abscesses	No, 16 months

Abbreviations.—BAL: bronchoalveolar lavage; GM: galactomannan; IS: immunosuppression; Tx: transplantation

### Direct spread from paranasal sinuses

**Fig 1:**
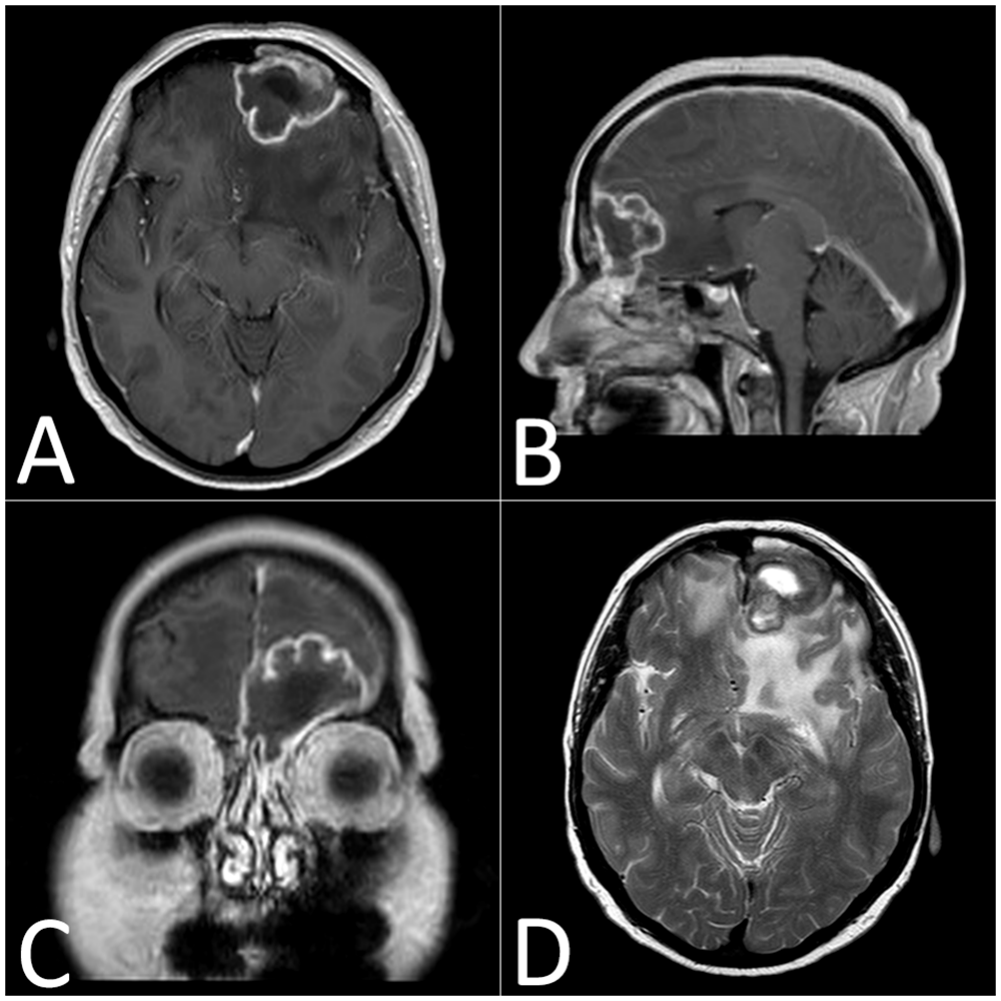
Multiplanar reconstruction of a frontal cerebral abscess with a frontal sinus starting point (patient #5) explored with gadolinium-enhanced T1W (A, B and C) and T2W (D). These sequences show a large frontal edema and a polylobulated abscess with a necrotic center and a peripheral annular enhancement.

In eight patients, the cerebral infection occurred during sinusitis (*n* = 6) or after a craniofacial trauma (*n* = 2). Only two of them were immunocompromised, the other six being immunocompetent. We identified nine parenchymal lesions. Five patients had a single lesion. T2*W imaging showed a significant peripheral low signal with susceptibility artifacts in favor of bleeding complications (*n* = 8). DWI highlighted the presence of one target-like ADC signal lesion in two patients (Table 3). MRI typically demonstrated a peripheral invasive lesion starting next to the nasosinusal cavities. Its outlines were polylobulated with a well-defined rim enhancement after gadolinium. The spreading by contiguity involved the concentric layers of the CNS from the dura mater to the white matter accompanied by a sizeable inflammatory phenomenon and a significant vasogenic edema (Fig 1). The progress of the abscess into the brain was accompanied by a subdural empyema in four patients. All patients underwent surgery and survived the infection with a follow-up of 7 months to 5 years.

**Table 3:** Numbers and type of lesions on initial MRI for the eight patients with direct spread of infection.

		Topography						
#/age/sex	No. of non- vascular lesions	Invasive parenchymal lesion	Subdural empyema	Hemorrhage	T2 Intermediate with surrounding high signal	Target-like ADC	Contrast enhancement Annular/Nodular	Vascular complications
**1 /59/M**	0	0	0	0	0	0	-/-	3
**2 /73/M**	2	1	1	1	0	0	1/0	0
**3 /72/M**	2	1	1	0	0	0	1/0	0
**4 /77/M**	5	4	1	4	0	0	4/0	1
**5 /55/F**	1	1	0	1	0	1	1/0	0
**6 /77/M**	1	1	0	1	0	0	1/0	0
**7 /65/F**	1	1	0	1	0	1	1/0	0
**8 /84/M**	1	0	1	0	0	0	0/0	0

### Hematogenous dissemination

**Fig 2:**
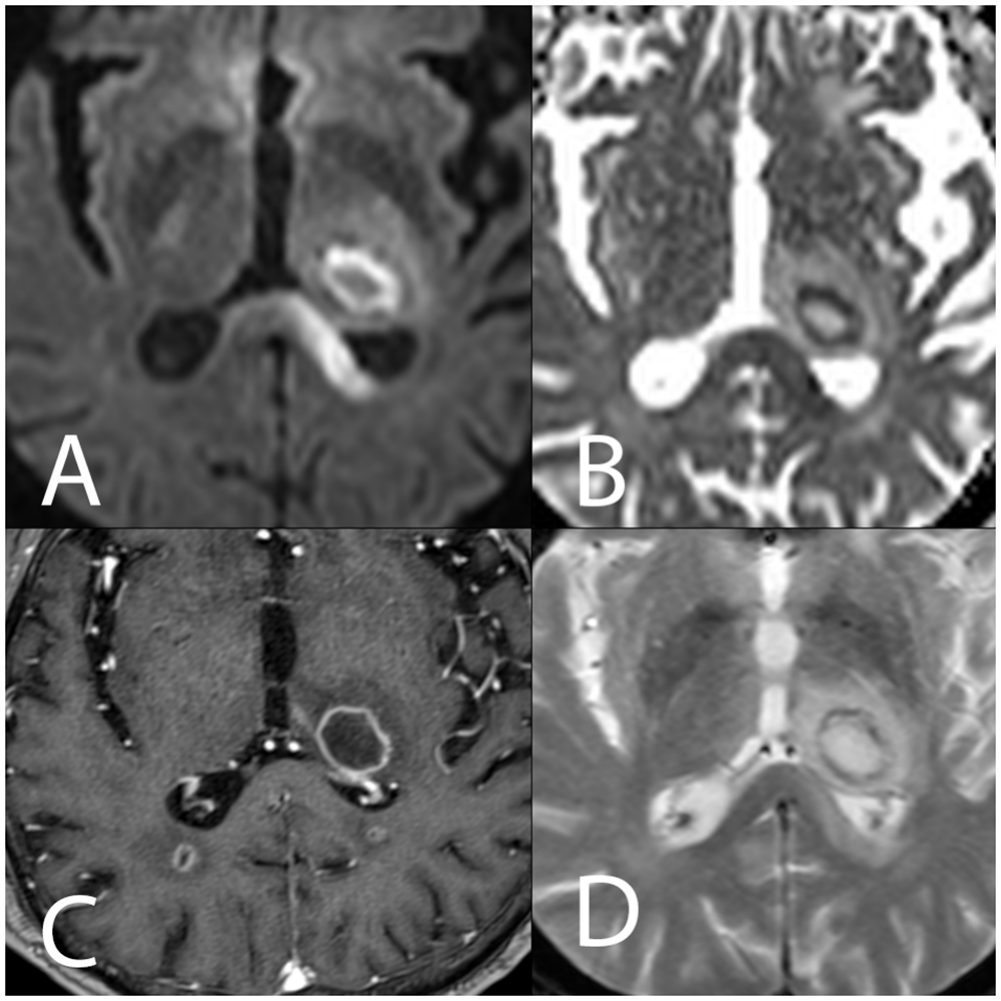
Left thalamic abscess with target-like characteristics (patient #10). The DWI sequence (A) and the ADC cartography (B) show a central hypointense area on DWI (high ADC value), a hyperintense circular area on DWI (low ADC) and a peripheral milder hyperintensity (upper ADC value rim). (C) Annular peripheral enhancement after gadolinium injection on T1W images. (D) Mild hypointense rim on T2*W images.

**Fig 3:**
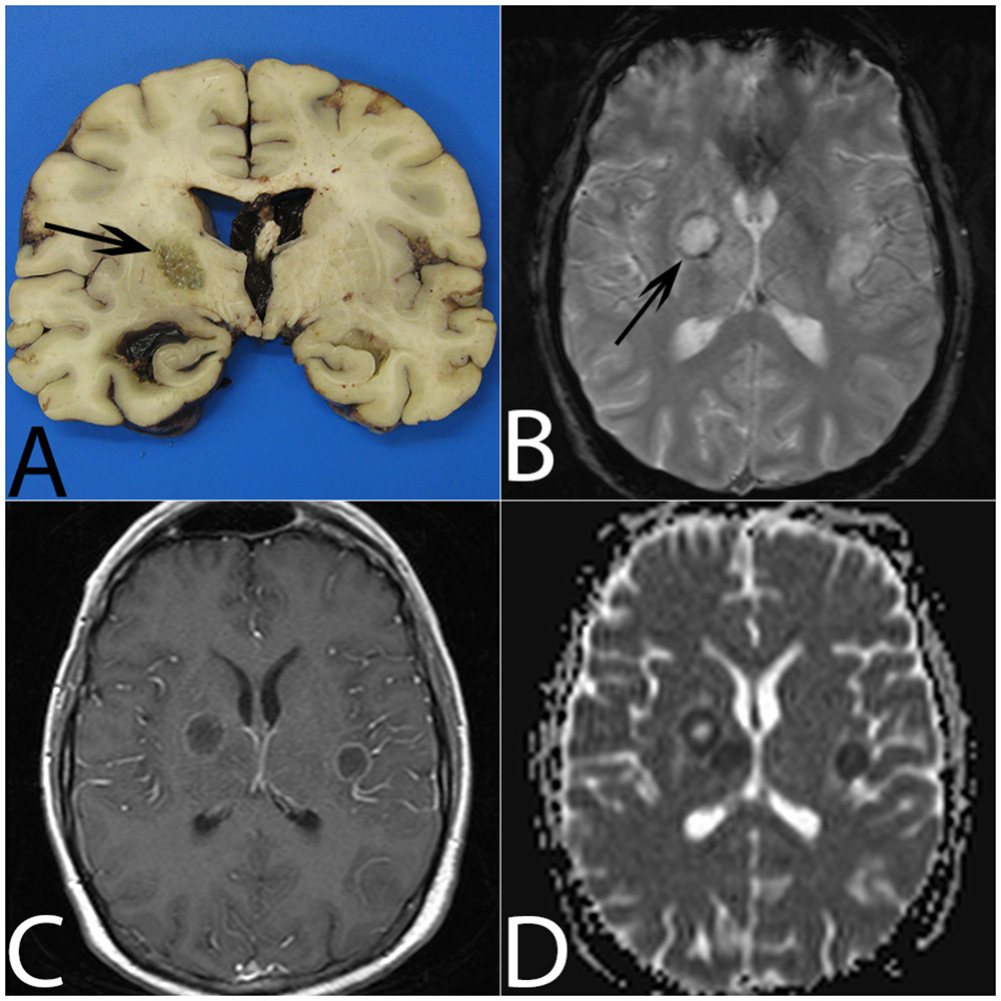
Aspergillosis abscess in the right thalamolenticular area due to hematogenous dissemination (patient #13). (A) On gross examination, the lesion is non-hemorrhagic with central necrosis (arrow). (B) On T2*, the abscess is surrounded by a mild hypointense ring (arrow). (C) Gadolinium-enhanced T1W imaging shows mild annular enhancement. (D) ADC cartography shows a target-like lesion with a central high ADC value, a circular area with a low ADC value and a peripheral upper ADC value rim.

Thirteen patients had a total of 329 parenchymal lesions (Table 4). All of these patients were immunosuppressed. The infection involved all location in the brain with a predilection for the corticomedullary junction in the supratentorial area (203/329, 62%), white mater (14%), basal nuclei-thalamus (9%) and infratentorial structures (7%). A corpus callosum lesion was seen in six patients. A hemorrhagic component was seen on T2*-weighted images as a significant hypointense signal in 42 lesions (13%). We noted contrast enhancing for 168 lesions (61%), with a more frequent annular shape (*n* = 96) compared to the nodular type (*n* = 72). A T2W intermediate signal with a surrounding high signal appeared for 26 lesions (8%) and 16 lesions for the target-like ADC signal (5%), with a minimum size of 9 mm (Figs 2 and 3).

**Table 4:** Numbers and type of lesions on initial MRI for the 13 patients with hematogenous dissemination.

		Topography										
#/age/sex	No. of non-vascular lesions	CMJ	BNT	CC	WM	BS	Subtentorial	Hemorrhage	T2 Intermediate with surrounding high signal	Target-like ADC	Contrast enhancement Annular/Nodular	Vascular complications
**9 /74/M**	18	11	4	1	0	0	2	3	0	0	0/0	0
**10 /64/M**	67	50	2	1	11	0	3	2	13	1	13/53	0
**11 /47/F**	47	26	4	2	5	1	9	7	0	1	32/15	1
**12 /35/M**	23	9	3	2	9	0	0	2	0	0	0/0	0
**13 /44/M**	18	9	2	0	3	2	2	4	0	1	14/0	8
**14 /57/M**	1	1	0	0	0	0	0	0	0	0	1/0	0
**15 /59/M**	44	33	1	1	4	1	4	6	0	0	0/0	0
**16 /58/M**	7	5	2	0	0	0	0	3	5	2	0/0	0
**17 /55/M**	6	5	0	1	0	0	0	3	0	1	2/4	0
**18 /53/M**	24	17	6	0	0	0	1	1	0	0	16/0	0
**19 /49/M**	6	5	1	0	0	0	0	4	4	6	6/0	0
**20 /58/F**	53	38	3	0	11	1	0	7	0	2	*NA*	0
**21 /58/M**	15	8	3	0	2	0	2	0	4	2	12/0	0
***TOTAL***	***329***	***217***	***31***	***8***	***45***	***5***	***23***	***42***	***26***	***16***	***96/72***	-

Abbreviations.—CMJ: corticomedullary junction; BNT: basal nuclei and thalamus; CC: corpus callosum; WM: white matter; BS: brainstem; *NA*: not available

Comparison according to the dissemination pathway showed that patients with hematogenous dissemination were younger (*p* = 0.003), more often had immunosuppression (*p* = 0.005), had more intracranial lesions (*p* = 0.0004) and had a higher 12-week mortality rate (*p* = 0.046) than patients with direct spread.

### Vascular complications

**Fig 4:**
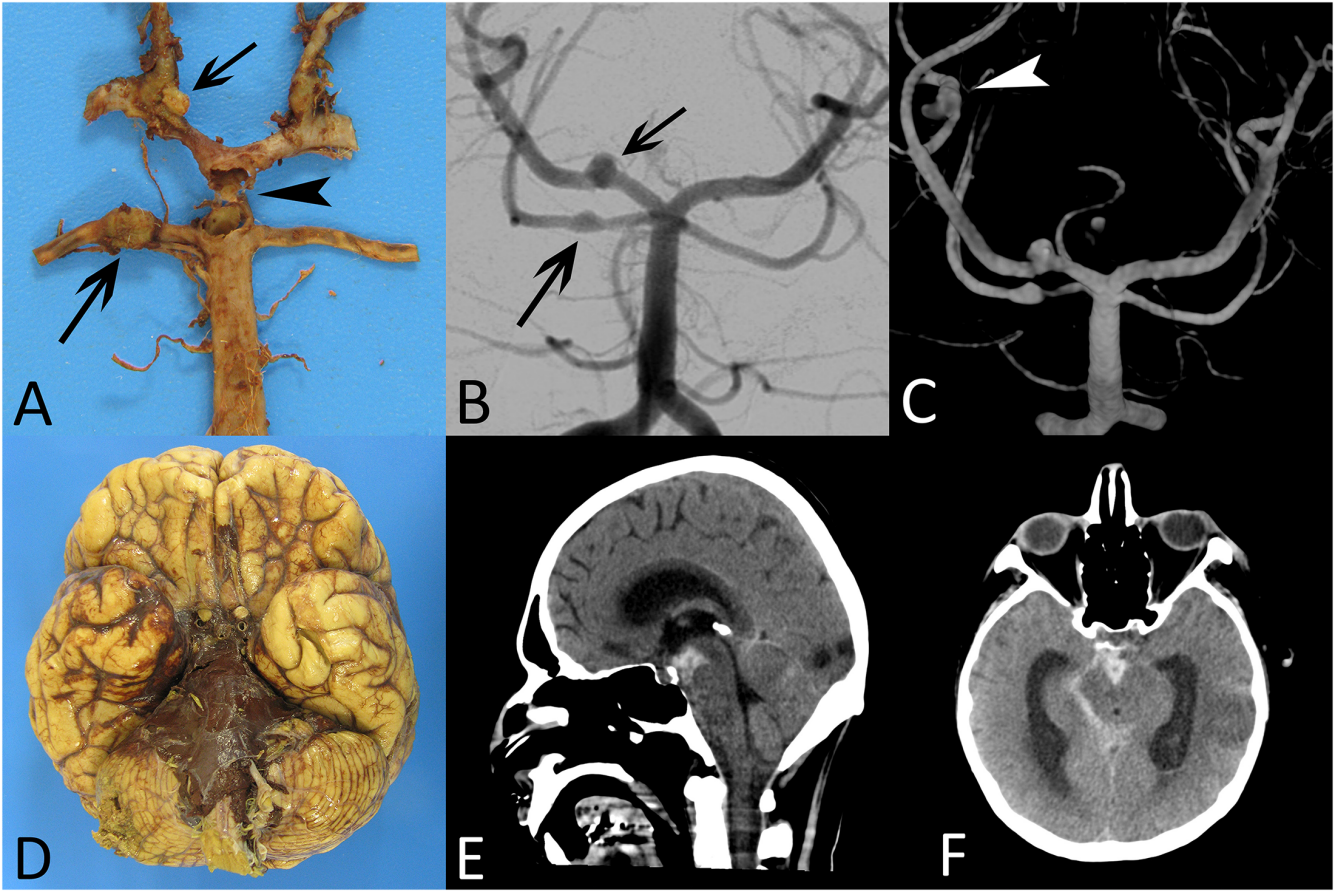
Macroscopic and imaging characteristics of vascular complications (patient #13). Gross examination (A) and cerebral angiogram (B) show aneurysmal lesions on superior cerebellar artery, posterior cerebral artery (arrows) and a ruptured aneurysm of the distal part of the basilar artery (arrowhead). The 3D angiography (C) shows an additional distal fusiform aneurysm on the middle cerebral artery (arrowhead). Massive cerebral hemorrhage into the basal cisterns (interpedoncular and pontine cisterns) visualized on gross examination (D) and non-enhanced CT scan (E and F).

Table 5 summarizes the main points concerning the vascular lesions. Two immunocompetent patients with direct spread presented macroscopic vascular complications. We identified three saccular arterial aneurysms with a proximal topography (internal carotid, sphenoidal segment of the middle cerebral artery, anterior communicating artery). Moreover, one patient developed a cerebral venous sinus thrombosis. Two immunocompromised patients with hematogenous dissemination had a total of nine aneurysms: six distal fusiform and three proximal aneurysms (two saccular and one fusiform). The death of these two patients was due to aneurysmal rupture causing a massive cerebral hemorrhage. Furthermore, the aneurysm of the distal part of the basilar artery causing death of patient #13 was not visualized on the imaging studies (CT, cerebral angiography) (Fig 4). This phenomenon could be explained by an aneurysmal collapse during the acute phase.

**Table 5:** Characteristics of vascular complications.

		Topography		
#/age/sex	No. of aneurysms	Proximal	Distal	Characteristics
**1 /59/M**	2	2	0	Thrombosis of lateral sinus
				Internal carotid artery / proximal / saccular
				Middle cerebral artery / proximal / saccular
**4 /77/M**	1	1	0	Anterior communicating artery / proximal / saccular
**11 /47/F**	1	0	1	Middle cerebral artery / distal / fusiform
**13 /44/M**	8	3	5	Superior cerebellar artery / proximal / fusiform
				Posterior cerebral artery / proximal / saccular
				Posterior cerebral artery / distal / fusiform
				Internal carotid artery / proximal / saccular
				Pericallosal artery / distal / fusiform
				Thalamic artery / distal / fusiform
				Middle cerebral artery / distal / fusiform
				Middle cerebral artery / distal / fusiform

### Gross pathology examination findings

Postmortem examinations were only performed on patients with hematogenous dissemination. These procedures revealed the presence of disseminated abscesses involving the whole brain with a predilection for the corticomedullary junction. In some cases, the autopsy identified hemorrhagic complications such as intralesional bleeding or subarachnoid hemorrhage. Arterial dissection revealed the presence of both proximal saccular and distal fusiform aneurysms.

### Microscopic examination findings

**Fig 5:**
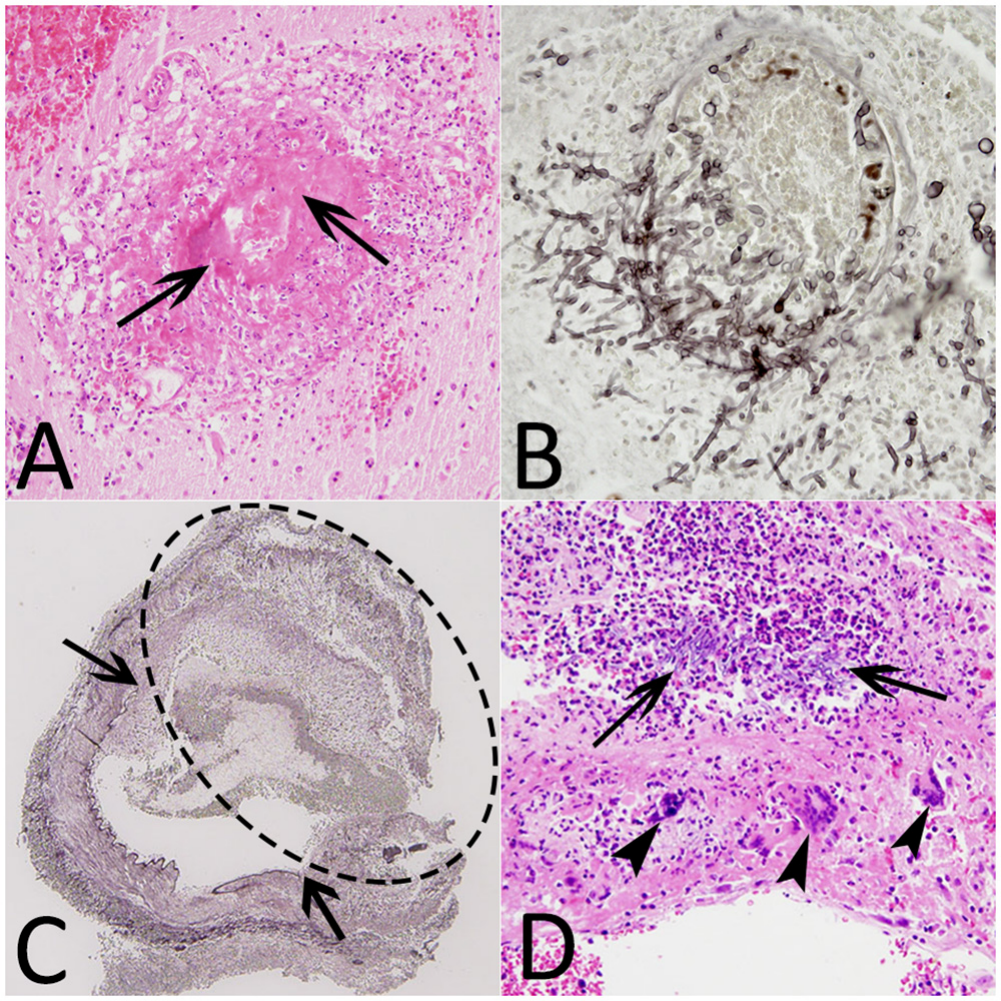
Histological findings (patients #9, #12, #13). (A) Hematoxylin-eosin stain (HE) (×20), destruction of a vessel with fibrinoid necrosis (arrows). (B) Grocott methenamine silver stain (GMS) (×40), vascular wall invaded by branching septate hyphae. (C) GMS (×4), intracerebral fungal aneurysm (dotted ellipse) with the interruption of the internal elastic lamina (arrows). (D) HE (×20), aneurysm wall containing hyphae, polynuclear cells (arrows) and giant cells (arrowheads).

**Fig 6:**
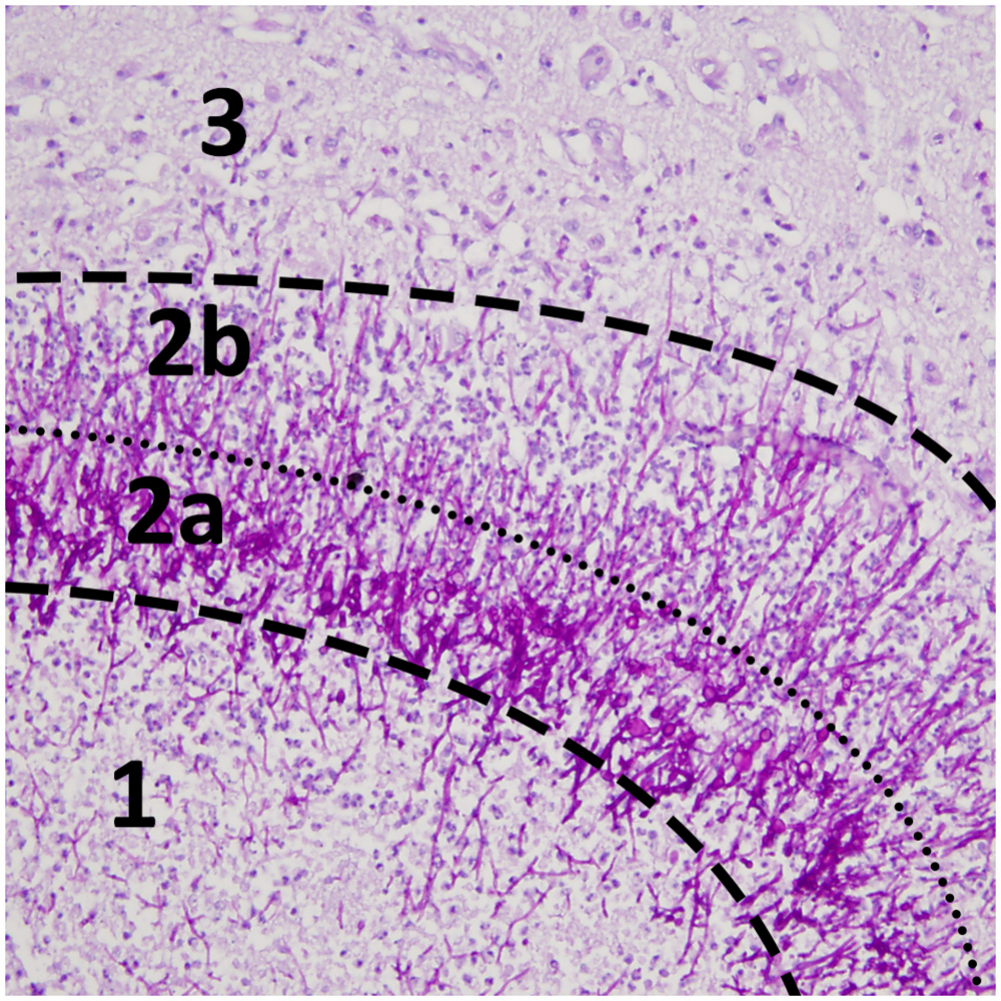
Histological abscess layers. Periodic acid-Schiff stain (×20) shows distinct areas with (1) central necrosis, (2a) an intermediate dense hyphal rim, (2b) an external layer of granulation tissue and (3) edematous brain tissue. On MRI, annular enhancement after gadolinium and mild hypointense signal on T2*-weighted images correspond to layer 2a and 2b (see Fig 2C and 2D).

The microscopic analysis of the vascular elements revealed that some vessels had been thrombosed by fungal invasion. Infected vessels showed wall destruction by hyphal invasion associated with an area of fibrinoid necrosis leading to red blood cell extravasation (Fig 5A and 5B). The aneurysmal wall was characterized by interruption of the internal elastic lamina and advanced destruction in which identification of the wall components (intima and media) was no longer possible. The pathologic wall was invaded by hyphae and inflammatory cells (granulocytes, giant cells) (Fig 5C and 5D). The most frequent lesions identified were abscesses. The microscopic examination highlighted three-layer lesions with a necrotic center, surrounded by a dense hyphal rim encircled by granulation tissue with or without giant cells (Fig 6). The abundance of granulocytes with or without giant cells is usually inversely related to the density of hyphae.

### MRI evolution in a long-term survivor

**Fig 7:**
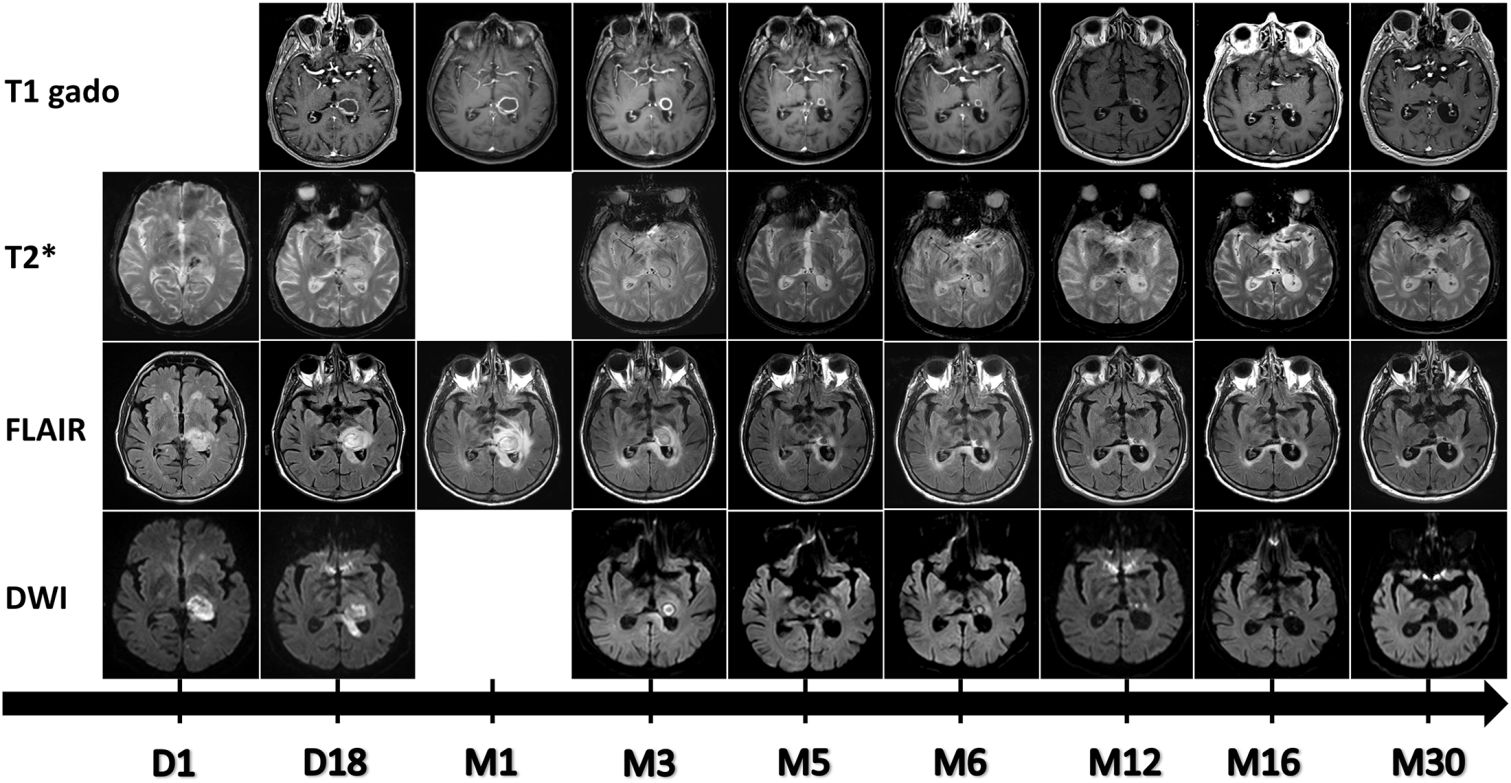
Evolution of a left thalamic abscess (patient #10) from day 1 to month 30. T1 after gadolinium injection, T2*, FLAIR and Diffusion-weighted images show annular peripheral enhancement with central necrosis and a progressive decrease in size starting at month 3.

Data from patient #10 who had sequential MRI assessments revealed persistent contrast enhancement after 30 months for the largest lesion (thalamic topography initially measuring 2.6 cm) (Fig 7). Originally, the enhancement was annular and progressed towards a nodular shape after 3 months. A majority of lesions disappeared after 6 months on DWI, which seems to be the most sensitive sequence. The lesions increased in number and size on MRI performed 2 weeks after start of voriconazole therapy. The smallest lesions evolved to complete disappearance, leaving a hypersignal on the FLAIR sequence. One of the corticomedullary junction lesions evolved to a punctiform calcification in a granulomatous process. The progression of the target-like ADC signal was marked by the disappearance of a central hypointense signal on DWI. For the largest thalamic lesion, we also noted a persistent DWI hypersignal over 1.5 years.

### Outcome

Eight (38%) of the patients died within 12 weeks. All were immunosuppressed and died of aspergillosis. Two immunosuppressed patients died beyond week 12, one of aspergillosis and one of another cause, 11 and 16 months after diagnosis respectively.

## Discussion

**Fig 8:**
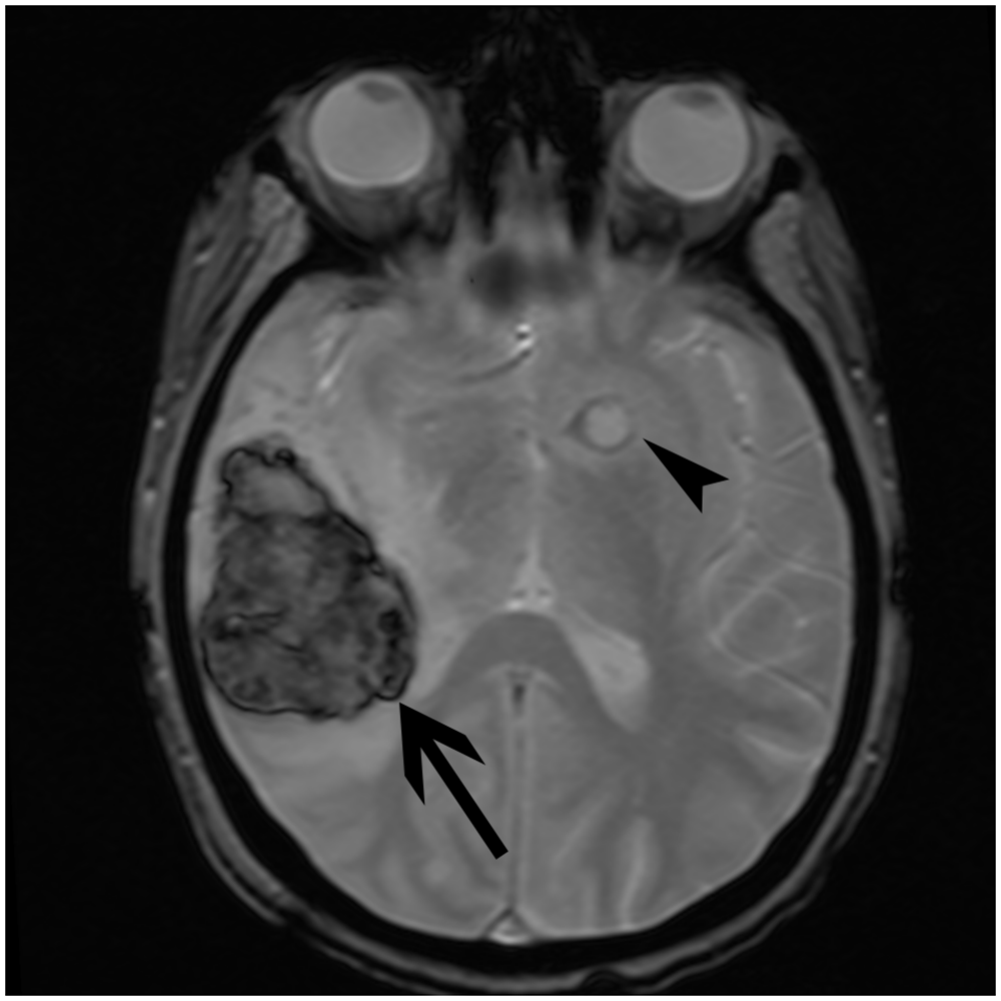
Hypointense signal T2*. Comparison between a hemorrhagic lesion (marked central and peripheral hypointensity areas) (arrow) and a non-hemorrhagic abscess (mild annular hypointensity) (arrowhead) (patient #11).

Cerebral aspergillosis has a poor prognosis. In our literature review on MRI aspects in cerebral aspergillosis (Table 1), based on studies published between 1994 and 2013, the mortality rate was 86%. In our patients, the 12-week mortality rate was surprisingly low (38%) with a significant difference in the six immunocompetent patients (0%) and in the 15 immunosuppressed patients (53%). Possible explanations for the improvement of survival in our series are the treatment with a voriconazole containing antifungal regimen, the use of surgery in nearly half of the patients and lack of immunosuppression in six (29%) of the patients. Voriconazole is now the gold standard for the treatment of IPA [27]. Its activity in cerebral aspergillosis has been demonstrated with only a death due to aspergillosis in only 37 patients (46%) out of 81 patients treated with voriconazole [28]. The critical role of surgery is also suggested in this study. Comparing different MRI sequences (including T1W, T2W, FLAIR, T2*W, T1W after gadolinium, and DWI), the investigations confirm that diffusion-weighted imaging appears to be the most sensitive modality for early identification of cerebral aspergillosis [12,13,16,18]. Direct spread of infection essentially begins from the paranasal sinuses. In accordance with the literature, a majority of the patients were immunocompetent [5,17,20]. The presentation is fairly similar between the different cases. MRI reveals a polylobulated abscess with peripheral contrast enhancement, substantial inflammation involving adjacent structures (paranasal sinuses, dura mater with focal meningitis, osteomyelitis) and a significant parenchymal edema. Subdural empyema may be associated. For the immunocompromised patients, cerebral invasion most commonly results from hematogenous spread. Histological investigations confirm the accepted mechanism consisting of an arterial occlusion by a septic thrombosis. The fungus passes through the wall of the blood vessel by producing elastase, which digests the internal elastic lamina and causes abscess formation. We observed distinctive lesions displaying central coagulative necrosis surrounded by a dense hyphal rim and a more external granulocyte layer. This physiopathology explains the topographic specificities. Indeed, lesions have essentially been seen at the corticomedullary junction, white matter, basal nuclei and thalami. These data can also be attributed to the vascular infection mode involving cortical arteries, which may be the first to lose their patency because of their narrower diameter [13]. The impairment of the basal nuclei and thalami is also characteristic and indicates the involvement of lenticulostriate and thalamoperforator arteries [5,6,11]. In our study, more than half of the patients had a corpus callosum lesion. This structure is involved in relatively few other processes (e.g., high-grade astrocytoma, cerebral lymphoma, multiple sclerosis and Marchiafava-Bignami disease), which can be readily differentiated from aspergillosis on the basis of their clinical and biological context [13]. Prior studies have assumed that contrast enhancement after gadolinium injection is correlated to the immune status in the sense that profoundly immunocompromised patients have no enhancement because of the absence of inflammatory response [4,6,12,21,29]. It is also accepted that a well-defined ring enhancement is quite uncommon [29]. Moreover, several researchers have suggested that the absence of enhancement seems to be a poor prognosis factor [6,23]. In our literature review, for 65 patients from comparable studies, 59 were immunosuppressed. Contrast enhancement was observed in approximately 55–60% of the patients [4–6,12–15,18,22]. The analysis of our data confirms that 61% of the lesions were enhanced in both the entire group and the immunocompromised group. Contrast enhancement is not correlated to the immune status because many profoundly immunosuppressed patients had well-defined enhancing lesions. In addition, none of our data suggest that the absence of contrast enhancement is associated with a higher 12-week mortality rate since half of the deceased patients showed enhancement (4/8 patients). Analysis of the T2- and T2*-weighted hypointense signal is difficult (Fig 8). Previous articles had addressed the issue, explaining that this signal could be the result of either ferromagnetic fungal deposits (iron, manganese, magnesium, zinc) [30] or the presence of methemoglobin (in the capsule wall and/or the macrophages) or the presence of free radicals produced by the macrophages [5]. Histologically, a mild T2*-weighted hypointense signal does not appear to be associated with a significant hemorrhagic phenomenon, although some red blood cells are associated with a significant amount of hyphal structures in this area. It is therefore difficult to separate these phenomena. To check whether a lesion is hemorrhagic, we had to rely on a qualitative criterion such as a significantly high hypointense signal. According to this, an estimated 13% of lesions in our study were hemorrhagic, in comparison to the literature data reporting a 25–39% rate [14,23]. Two signal features on MRI could be more specific of a cerebral *Aspergillus* abscess. Firstly, Miaux *et al*. described a central intermediate signal on T2-weighted sequences, corresponding to coagulative fungal necrosis at autopsy findings [4,12]. This feature is surrounded by a high hyperintense signal corresponding to vasogenic edema and accounted for 8% of the total lesions. Secondly, the present investigation confirms the presence of target-like ADC signal lesions, accounting for approximately 5% of the lesions. In immunocompromised patients, ring lesions on DWI occur mainly in cerebral aspergillosis but also in progressive multifocal leukoencephalopathy and CNS lymphoma [31]. We could juxtapose this target-like imaging element to the histological findings and particularly the characteristic three-layer lesion, which could explain the signal modification (Figs 2 and 6). Central necrosis presents two components responding differently on DWI. This feature is probably due to a difference in density impacting the Brownian motion of water molecules. The intermediate layers composed of a dense hyphal rim and peripheral inflammatory hypercellularity are visualized by contrast enhancement and a mild hypointense signal on magnetic susceptibility sequences (T2*W) but has no match on DWI because of an insufficient spatial resolution. Mycotic aneurysms historically have accounted for 2–4% of all intracerebral aneurysms. *Aspergillus* is the most common agent that causes fungal aneurysms. It is considered that these lesions have a high risk of rupture [32]. Our results suggest that the two spreading pathways should be distinguished. The direct spread from paranasal sinuses produces saccular aneurysms on the proximal arterial portion from the circle of Willis. This process starts from the external vessel surface, penetrates the vasa vasorum system and gradually affects all layers including the adventitia, which leads to the weakening of the wall and subsequent aneurysm formation [17,20,33]. Conversely, the hematogenous dissemination in immunocompromised patients more frequently leads to fusiform aneurysms with a distal topography. In this context and compared to the literature data, our two patients (patients #11 and #13) are rare examples of fungal aneurysms without sino-nasal involvement. The follow-up of a long-term survivor has raised some interesting points. Firstly, this patient had the highest number of lesions, which could suggest that the extent of the disease may not be correlated to mortality. The only poor prognosis factor appears to be advanced immunosuppression. Secondly, the largest residual cerebral lesion, located in the thalamus, was still enhancing 2.5 years after onset of the disease. Such long-term contrast enhancement has already been described by Adler *et al*. regarding a basal ganglia fungal lesion on an enhanced MRI study obtained 3 weeks after the onset of neurologic symptoms [4,34]. Thirdly, the comparison of the first three MRI performed showed that lesions may increase in number and size even after 2 weeks of effective treatment with voriconazole. Fourthly, the initial examinations show annular contrast enhancement for almost all lesions. Progression will be towards nodular enhancement as the lesion regresses. In general, the progression of the disease is marked by the complete disappearance of the smallest lesions leading to a residual FLAIR hypersignal relating to a gliosis phenomenon. For the majority of lesions, the DWI hyperintense signal remains present for approximately 6 months. However, we have noted the presence of a lesion evolving like a granulomatous structure leaving a calcified scar. On the necropsy from another patient, a giant-cell granuloma was identified and could explain this type of imaging course. This last issue has been reported by Ashdown *et al*., among others, stating that calcifications likely represent healing or healed granulomas and are seen more often if the patient survives the acute infection [5,13,29].

## Conclusion

Cerebral invasion during aspergillosis is a severe and often fatal complication. To increase the chances of survival, physicians need to diagnose infection earlier to introduce rapidly an effective antifungal treatment. MRI appears to be the exam of choice and should require at least T1W, T2W, FLAIR, T2*, DWI and T1W after gadolinium injection sequences for diagnosis and follow-up.
